# Grieving for Job Loss and Its Relation to the Employability of Older Jobseekers

**DOI:** 10.3389/fpsyg.2019.00366

**Published:** 2019-02-26

**Authors:** José Antonio Climent-Rodríguez, Yolanda Navarro-Abal, María José López-López, Juan Gómez-Salgado, Marta Evelia Aparicio García

**Affiliations:** ^1^Universidad Isabel I de Castilla, Burgos, Spain; ^2^Department of Social, Developmental and Education Psychology, Faculty of Education Science, University of Huelva, Huelva, Spain; ^3^Department of Clinical and Experimental Psychology, Faculty of Education Science, University of Huelva, Huelva, Spain; ^4^Department of Nursing, University of Huelva, Huelva, Spain; ^5^Espíritu Santo University, Guayaquil, Ecuador; ^6^Department of Social, Work and Differential Psychology, Faculty of Psychology, Complutense University of Madrid, Madrid, Spain

**Keywords:** grieving, unemployment, employability, older workers, work centrality, availability, perceived difficulties, fears

## Abstract

**Introduction:** Loss of employment is an experience that is lived and interpreted differently depending on a series of individual variables, including the psychological resources available to the affected person as well as their perception of their degree of employability. Losing one’s job can be one of the most painful and traumatic events a person has to withstand. Following a dismissal, the worker needs to overcome a period of emotional adaptation to the loss. But that period of grieving can also condition the job searching process of the individual and can be influenced by different variables, highlighting the age and work experience. The objective of this study is to analyse the relationship between intensity and type of affliction due to the loss of employment in older workers and their level of employability.

**Methods:** We carried out a descriptive and analytical cross-sectional study. The sample consisted of 140 unemployed participants, from 19 to 65 years of age—users of Job Orientation in the Public Employment Service of Andalusia (Spain). Of the total participants, 66 were unemployed and over 45 years of age. They all took the Labour Insertion Potential Assessment Test and the Texas Revised Inventory of Grief, adapted for job loss.

**Results:** Significant differences are shown in the grieving process due to loss of employment between both groups, with the older unemployed living the process more intensely. In relation to the employability potential, differences are found between both groups in terms of availability, perceived difficulties and fears. Interrelationships between total grieving intensity and the importance that older jobseekers give to work are also indicated.

**Conclusion:** Loss of employment and the psychological and health consequences of this situation are identified with those that arise in the grieving process. Older workers present a series of features that determine that their job loss grieving process is more intense and lasts longer than that of other younger workers, regardless of whether the job loss was recent or not. On the other hand, it is shown that the intensity of grieving for job loss is related to the decrease of certain variables that are part of the concept of employability.

## Introduction

The term grieving can be applied to those psychological and psychosocial processes that come into play in the face of any type of loss or change relevant to the person who suffers them, such as academic failure, divorce, family issues, changes of address, economic problems, diagnosis of serious illness, migratory movements, empty nest syndrome or retirement. All these situations can lead to maladaptive reactions with manifestations such as sadness, crying, despair, impotence, anger and guilt, as well as social and workplace dysfunction (Carmona et al., 2008; Cáceres et al., 2009; Shear et al., 2013).

Job loss triggers a process of global adjustment in the person in all their personal, social and family dimensions (Afonso and Poeschl, 2006), and involves losing contact with a situation with which an affective bond is maintained. In this way, an expression similar to that of mourning in any other circumstance takes place, such as that characterising the loss of a loved one (Karsten and Moser, 2009; Buendía, 2010).

The several explanatory theories of grief range from those that emphasise the role of separation anxiety in the process (Munera, 2013), through those that conceive a biological perspective of grief by placing it as a consequence of attachments (Bowlby, 1980), to those focused on analysing the processual nature of grief, with a beginning, development and end (Ortego-Maté, 2001). Several authors have presented models in which the phases of grief are identified as a process (Kübler-Ross, 1993; Sánchez and Martínez, 2014). Other authors (Fernández et al., 2006), however, criticise this passive view of grief by phases, and understand that coping with grief calls for an active attitude. These models state that grief is influenced by multiple causes, both individual and social, and this makes it very difficult to determine its stages. Another of the issues approached in the literature on grief is related to its typologies. Neimeyer (2002), in addition to normalised grief, distinguishes between anticipatory grief, felt before the loss; inhibited grief, with pathological denial of loss; prolonged grief, when the sufferer tries to maintain that the lost person or situation is still “alive”; and complicated grief, accentuating and prolonging the typical processes of normal grieving.

Dysfunctional grieving processes favour somatisation and do not allow its evolution (Villacieros et al., 2014; Arizmendi and O’Connor, 2015). Possible risk factors for these types of specifically dysfunctional grief have been defined; whether the loss is expected or unexpected (Sanders, 1999); the economic impact of the loss (Hegewald and Crapo, 2007); strategies and styles for coping with the loss (Siracusa et al., 2011; Tofthagen et al., 2017); and other possible related sociodemographic variables as modulators of the use of different coping strategies in the face of loss (Meléndez et al., 2012).

Regarding protection factors, as indicated by Stroebe et al. (2006), the most notable are religious beliefs and social support (Álvaro and Garrido, 2003), resilience (Gillham and Seligman, 1999; Yu et al., 2016), post-traumatic growth and strong personality or hardiness (Tedeschi and Kilmer, 2005).

Díaz et al. (2016) carried out a study in which they applied the Texas Revised Inventory of Grief (TRIG) for the first time in a job loss context. The participants in this study, a group of unemployed Spaniards, did not present differences in the intensity of grief due to job loss related to age and sex, but in relation to the length of unemployment and responsibility for family income. On the other hand, the study evidenced avoidant coping in people who presented more intense grief.

Conceptualising employability is not an easy task, as the term is used in the employment and unemployment area to refer to various issues related to greater or lesser ease of entering the labour market (Gamboa, 2013). Tackling it through different disciplines, approaches and levels determines the study perspective and, finally, its meaning and explanatory scope (Rentería-Pérez and Malvezzi, 2008).

The International Labour Organisation defines it as the likelihood of filling a vacancy in a specific job market based on the attributes with which the searcher is equipped, and which are those that allow this individual to overcome the obstacles imposed by the market (International Labour Organisation, 2000). Other authors have placed more emphasis on the psychosocial approach to conceptualising employability, indicating it as the degree of suitability of the psychosocial characteristics of a job seeker for the typical profile of the person employed in a given context (Blanch, 1990). In terms of the latter approach, employability therefore includes qualifications, knowledge and skills that increase the workers’ ability to find and keep a job. In short, a series of internal factors or psychosocial factors that affect the attitudes and motivation of the unemployed are highlighted which are related to the decrease in their chances of finding a job. Thus, as Blanch (1990) explains, it seems that the “psychosocial profile” of the unemployed has a significant influence on their access to employment and determines their level of employability.

The concept of employability is multidimensional, related to and being influenced by different variables (McQuaid and Lindsay, 2005; Rothwell and Arnold, 2007; Clarke, 2008; Rodríguez-Espinar et al., 2010; Törnoos Née Kirves et al., 2017). Although there are many proposals regarding the most significant variables that determine an individual’s employability, most of them point to work centrality, values and work goals, availability, style and difficulties in finding a job, attitudes and fears about the unemployment situation and the search for a new one, as well as the level of self-esteem, self-concept and self-efficacy in the person seeking employment as the most relevant (Alonso-García, 2010).

Work centrality has been defined as the degree of general importance that working has at any given time for an individual (Mejía-Reyes, 2016). Valls and Martínez (2004) distinguished two components in work centrality: the belief of work as a life role and as a working activity of the current moment in relation to other activities.

Moreover, it is important to highlight the distinction between two related concepts such as values at work and working goals or objectives. Values at work identify the basic reasons why people work, and work goals refer to what the person prefers to find in their work—i.e., their preferences about what they want to get from work. Thus, goals are more specific than values, despite having a similar meaning, as it is accepted that values do not directly influence people’s activity, but rather act indirectly through attitudes and goals (Alonso-García, 2004a).

Availability could be understood as the predisposition of the unemployed person as a conditioning factor in the search for work. Due to possible concurrent circumstances in the person and—usually—derived from their closest relational context, at that time, the unemployed person is or is not immediately available to start a job. The appearance of these circumstances will modulate the value that the person gives to finding a job. Consequently, this psychosocial factor is situated in the same dimension as work centrality, explaining part of the pressure, toward the search, that the individual perceives in their immediate environment (Martínez et al., 2001). In relation to the job search, the higher the jobseeker’s availability, the greater their chances are of finding a job. In contrast, when the limitations exceed what would “normally” be logical, they become a serious obstacle to labour insertion.

The individual’s perception regarding the difficulties or obstacles in their jobseeking process is also a variable closely related to their employability. The difficulties are the individual’s beliefs in their greater or lesser chances of finding work, and although they do not conform to reality, they may have a certain reactive predictive value (Wang et al., 2017). Fernández and Aramburu (2000) related these perceived difficulties within the unemployed to self-confidence. Aguiar and Bastos (2018) discussed the importance of self-concept and self-confidence for vocational choice. The levels of importance for self-confidence, self-esteem and self-concept in the search for employment have been considered as variables that may well be related to the difficulties perceived by the unemployed person in their jobseeking process (da Motta Veiga and Gabriel, 2016; Kakoudakis et al., 2017).

In their work on analysis and intervention in job search techniques, Aramburu and Fernández (1994) demonstrated that the reduction or extinction of fears and worries that may exist in relation to the search for employment in unemployed people increases expectations and diminishes fears. According to Alonso-García and Sánchez-Herrero (2011), there are different fears in the jobseeking situation that are negatively related to success in the search.

Gradual ageing of the workforce in industrialised countries in recent years has led to continuous growth in the number of older employees, which is a change compared to what happened in previous recession periods (Peiró et al., 2013). This trend, however, does not seem to occur in the countries of so-called Mediterranean Europe (Spain, Portugal, Greece, etc.), where the consequences of the crisis and labour changes continue to oust older workers from the job market (Alcover et al., 2014). Older workers are the main victims of the changes undergone in the job market as a result of technological and organisational breakthroughs (Izquierdo et al., 2014), making them one of the most vulnerable groups in terms of losing their jobs and with greater difficulties to re-enter the labour market, particularly suffering the negative effects of unemployment on their health and well-being, and predisposing them to stress situations prior to retirement (Villamil et al., 2006).

Many older workers who lose their jobs become “stable destabilised” (Arnal et al., 2013), immersed in an exclusionary job market that denies them the value of experience, relegating them within their own employment to underemployment or directly dispensing with them. These job loss situations in older workers are preceded by processes of adaptation to the new situation, obliging them to structure new routines, habits, identities, etc., that were already entrenched (Durbar, 2002). This means a change of life and a new way of facing the environment, which also requires redefining processes for one’s own personal identity (Amber and Domingo, 2017).

But this job loss situation is experienced differently by each worker on the basis of other variables (Zacher and Schmitt, 2016), with the different strategies deployed by the individual to understand their new situation playing an important modulating role (Demerouti et al., 2014; Segura and Topa, 2016).

The aim of this study is to analyse the relationship between the intensity and type of grieving due to job loss in older workers and their level of employability, assuming that those workers who have been able to develop more functional grief have a more suitable level of employability, with job loss grieving thus constituting a variable that modulates the employability of the person seeking employment. Another objective pursued in this study consists of comparing the intensity and type of grieving due to loss of employment in older workers with those in other groups of workers, and their relationship with employability levels, analysing possible age-related differences.

## Materials and Methods

### Participants

The sample consisted of a total of 140 unemployed participants (57 men and 83 women) who were attending an Employment Guidance Programme of the Public Employment Service of Andalusia (Spain) and whose ages ranged from 19 to 65 years (*M* = 43.03, *SD* = 12.75). Of the total number of participants, 66 were unemployed persons over 45 years of age (37 men and 29 women), of which 15 were over 56 years old. Regarding the level of studies in the sample, the majority of unemployed persons had primary (54.30%) and secondary (29.30%) studies.

### Instruments

All the participants in this study completed the following assessment instruments:

1. Sociodemographic data protocol: prepared ad hoc for this research, to gather information on the following variables: age, sex, education level, length of unemployment, dependents, receipt of unemployment benefit, etc.
2. Texas Revised Inventory of Grief adapted to the Job Loss Situation (Díaz et al., 2016): an adaptation of the TRIG, an instrument originally designed to assess the intensity of grief for the loss or death of a loved one, performed in the context of job loss (Faschingbauer et al., 1987). It consists of a total of 21 statements with a five-point Likert-type response format (from completely true to completely false) grouped into two dimensions or scales: Past grief and Current grief. The former (items 1 to 8) reports on the behaviour and feelings of the person at the immediate time of job loss, while the latter (items 9 to 21) refers to the feelings experienced at the current time in relation to said loss. With adequate reliability indexes in the adaptation to unemployed population (0.85 and 0.90 in the respective scales), the Cronbach’s alpha coefficients obtained in this sample were 0.81 and 0.88, respectively.
3. Labour Insertion Potential Assessment Test (Alonso-García, 2010): an instrument that evaluates people’s workplace insertion potential or employability. It consists of five independent scales that provide information on Work Centrality, Working Goals, Availability, Difficulties and Fears in jobseeking.
4. Centrality: from the adaptation carried out by Alonso-García (2004a) of the MOW International Research Group Questionnaire (1981, 1987), this comprises two subdimensions, the first of which rates the Absolute Importance of the work/job [Likert scale format response from 1 to 5 (from minor to greater importance)] and Relative Importance (answer scale format with three response alternatives), and Absolute Centrality around work [Likert scale response format from 1 to 7 (from least to greatest importance)]. In turn, in the second subdimension the participants had to point out the importance (from 1 to 5) of work/the job/employment compared to other facets of life (spouse/partner, family, friends, religion, hobbies, studies and volunteering), as well as proceeding to organise these aspects according to the importance given to them at the current time.
5. Work goals: this is an adaptation by Alonso-García (2004a) of the Salanova (1991) Scale, devised to measure the main objectives or goals that people prioritise when doing a job; that is, their preferences about what they want to get from work. It includes 14 items with a five-point Likert response format, grouped into five subscales: Independence at Work, Personal Development, Professional Development, Comfort and Instrumental Index. With a Cronbach’s alpha of 0.74 (Alonso-García, 2004a), the internal consistency achieved in this study was 0.71.
6. Availability: the participants had to indicate to what extent (on a scale of 1 to 5) they would be willing to accept a series of working conditions. For a person to work in a certain occupation and stay in it, among other things, they must want to and be able to work; the sum of both aspects reflects what is designated availability. The scale consists of a total of 24 elements grouped into the following subdimensions: Geographical availability (DGE), Availability for physical effort (DEF), Availability on schedules (DHR), Jobs or tasks that I do not like (APN), Unattractive conditions (ACP) and Sales (AVE). With a reliability of 0.79 (Alonso-García, 2004b), in this study the alpha coefficient obtained was 0.75.
7. Difficulties: this scale refers to possible obstacles or setbacks perceived by the person when looking for a job. Composed of 24 elements; the person must indicate the level of difficulty perceived (from 1 to 5) for each of them. In turn, it is subdivided into five subscales: Family Responsibilities (IRF), Training (IFO), Attributes (IAT), Personal Characteristics (ICP) and Jobseeking Skills (IHB). The instrument provides a final open response item. With a Cronbach’s alpha of 0.85 (Alonso-García, 2003), the reliability obtained in this work was 0.82.
8. Fears: a scale composed of different situations that can be a source of fear in the jobseeking process. With a Likert-type five-point response format, the person points out to what extent or how intensely (from 1 to 5) they generate fear. It consists of 29 items grouped in turn in six dimensions: Fear of not measuring up (ENT), Fear of working hard, without a schedule and under rigid rules (ETM), Fear of selection processes (EPS), Fear of not being able to develop my talent at work (EDT), Fear of not knowing the future (EDF) and Fear of making a bad impression (EQM). The instrument provides a final open response item. With a total reliability of 0.93 (Alonso-García and Sánchez-Herrero, 2011), the alpha coefficient obtained in this study was 0.89.

### Procedure

The sample was obtained through the Public Employment Service of Andalusia (Spain). Specifically, all the people who—from January to June 2018—attended a scheduled appointment with the Labour Orientation Service at centres in the province of Huelva, were explained the objectives of the research and asked for their collaboration.

Those who voluntarily agreed to take part (68% response rate), were taken to a separate room where, after signing the corresponding informed consent form guaranteeing the anonymity and confidentiality of their data, as well as its use exclusively for research purposes, they filled in the assessment instruments individually in the presence of their reference counsellor, who would have been trained to administer the tests.

#### Ethical Approval Procedures

The study was conducted in accordance with the Declaration of Helsinki and the procedure for carrying out this research work was analysed and ratified by the Provincial Commission of the Andalusian Employment Service (SAE).

This procedure was carried out through the SAE, in whose facilities this research was carried out. This Commission is a dependent body of the regional government of Andalusia and maintains professional relations with the University. It operates as an Institutional Review Board that ensures the proper functioning of the public institution and performs, among other functions, the planning, management, promotion and evaluation of the different programmes and actions for employment in Andalusia. In particular, it is responsible for monitoring and evaluating the activities of the SAE Agency and proposing the measures it deems appropriate to ensure good praxis as well as ethical and deontological adequacy.

As the Commission was in charge of reviewing and approving this research, the University of Huelva, the institution of the authors, did not act as an assessment committee, nor did it require its own approval for the development of this project. The researchers signed their respective documentation before the members of the Provincial Commission of the Andalusian Employment Service (SAE) to ensure their commitment to the anonymity of the sample and respect for the participants’ rights.

Likewise, inclusion in the study was engaged ensuring the willingness to participate, full information about the process and the confidentiality of the interviewers. To this end, the consent obtained from the interviewees was both informed and written to ensure the correct ethical procedure.

### Data Analysis

The data analysis was carried out with the statistical package SPSS 22. First, the internal consistency of the assessment instruments administered was estimated, obtaining the Cronbach’s alpha reliability coefficients. We then performed a univariate analysis of the research variables, calculating central trend and dispersion statistics for the continuous variables, as well as frequencies and percentages for the categorical variables. For the comparison of metric variables, after checking normality by way of the Kolmogorov–Smirnov test, bivariate analyses were carried out, specifically Student’s *t*-test for independent samples. Finally, Pearson’s r correlation coefficient was determined in order to analyse the intercorrelations between the study variables.

## Results

In relation to the results obtained in this work, Table 1 first shows the descriptive statistics (means and standard deviations) of the variables studied in this work, as well as the corresponding comparisons of means carried out between older unemployed people and those under 45. As shown in this table, statistically significant differences appear in the job loss grieving process between both groups, with the elderly unemployed living the process with greater intensity (*t* = -15.599; *p* = 0.000). Likewise, they also experience a more intense grieving process both immediately after losing the job and at present, with statistically significant differences in both cases (*t* = -14.639; *p* = 0.000 and *t* = -15.023; *p* = 0.000, respectively).

**Table 1:** Descriptive statistics of the variables and comparisons of means between groups.

	Age group						
				
	Below 45 years (*n* = 74)		Over 45 years (*n* = 66)		t	gl	p
					
	M	*SD*	M	*SD*			
**Centrality**							
Absolute importance	3.08	1.39	1.73	0.89	6.93	125.36	0.00
Relative importance	1.99	0.91	2.73	1.07	-4.41	138	0.00
Absolute centrality	4.96	0.63	6.32	0.77	11.37	125.92	0.00
**Work goals**							
Partner	4.32	1.21	3.97	1.33	1.65	138	0.10
Family	3.58	1.22	4.68	0.73	6.58	121,29	0.00
Friends	3.80	1.17	3.86	1.13	-0.34	138	0.73
Work	4.58	0.76	4.62	0.55	-0.35	138	0.72
Religion	2.15	1.32	2.21	1.35	-0.28	138	0.78
Studies	3.09	0.76	4.50	0.66	-11.59	138	0.00
Hobbies	3.42	1.10	3.59	1.12	-0.91	138	0.36
Volunteering	2.92	1.23	2.91	1.44	0,04	128.77	0.97
**Availability**							
Total	77.35	12.83	78.98	14.36	-0.71	138	0.48
DGE	15.36	7.65	12.98	6.43	1.98	138	0.05
DEF	11.74	2.45	11.21	3.09	1.13	138	0.26
DHR	14.23	2.95	13.59	3.82	1.10	121.89	0.27
APN	4.46	1.48	8.42	1.41	-16.14	138	0.00
ANR	15.93	2.71	15.07	3.00	1.77	138	0.08
ACP	8.12	2.18	11.32	2.22	-8.59	138	0.00
AVE	7.50	2.19	6.38	2.50	2.83	138	0.00
**Difficulties**							
Total	48.46	20.60	110.50	216.22	-2.32	66.08	0.02
IRF	7.10	2.88	13.17	17.36	-2.80	68.28	0.01
IFO	10.81	15.80	9.64	3.21	0.59	138	0.55
IAT	8.65	3.05	27.17	56.90	-2.76	136	0.01
ICP	13.39	5.23	41.89	134.80	-1.72	65.17	0.09
IHB	9.05	3.66	18.64	36.98	-2.10	66.13	0.04
**Fears**							
Total	75.22	22.69	69.46	22.42	1.49	135	0.14
ENT	18.36	9.37	18.30	8.30	0.04	138	0.97
ETM	14.27	2.09	7.82	2.94	14.77	115.91	0,00
EPS	11.69	4.34	12.20	4.77	-0.66	136	0.51
EDT	7.47	3.19	7.95	3.90	-0.79	123.83	0.43
EDF	16.70	6.25	17.38	5.81	-0.66	138	0.51
EQM	6.72	3.65	6.14	3.39	0.97	138	0.33
**Grief**							
Total	39.08	13.13	81.54	18.54	-15.90	138	0.00
Past	14.27	4.84	30.64	7.85	-14.64	105.76	0.00
Recent	24.81	9.16	51.27	11.34	-15.25	138	0.00


*M, mean; SD, standard deviation; t, test T; gl, degrees of freedom; p, level of significance.*

Moreover, in relation to the potential for workplace insertion or employability, expressed through the variables Work Centrality, Goals, Availability, Difficulties and Fears in the jobseeking process, the results obtained were as follows: Regarding the first variable, Work Centrality, the data indicate the presence of significant differences in terms of absolute (*t* = -11.3711, *p* = 0.000) and relative centrality (*t* = -4.406, *p* = 0.000). In this sense, it is the older unemployed who attribute greater importance to work overall. Regarding the relative centrality, for the older group, the most important areas of life are, in this order, family, work, studies and life partner, whereas in the rest of the unemployed participants work appears as the most important area, followed by the life partner, friends and family. In contrast, no differences are detected in terms of preferences (work goals) about what both groups want to obtain from work.

Likewise, for Availability, a relevant variable that influences the acceptance or rejection of certain types of job as shown in Table 1, we observed statistically significant differences in four of the subdimensions considered of this variable. On one hand, Geographical Availability (*t* = 1.998; *p* = 0.048) and Sales (*t* = 2.829; *p* = 0.005), with older jobseekers in both cases stating to a lesser degree that they would not accept jobs that involved those working conditions. And, on the other hand, Jobs or tasks I don’t like (*t* = -16.140; *p* = 0.000) and Unattractive Conditions (*t* = 8.593; *p* = 0.000), working situations that in this case would be accepted to a significantly greater extent by the older unemployed.

Regarding the difficulties perceived in general by the participants in jobseeking, statistically significant differences are also observed between both groups (*t* = -2.424; *p* = 0.017), with older unemployed people clearly perceiving greater obstacles to their rejoining the labour market owing to their family responsibilities (*t* = -2.824; *p* = 0.004), their attributes (*t* = -2.640; *p* = 0.010), or their jobseeking skills (*t* = -2.096; *p* = 0.040).

Finally, in relation to the fears perceived by unemployed people in the jobseeking process and related situations, the results show that statistically significant differences only appear between older unemployed people and the rest of the workers in the factor Fear of hard work, with no schedule and strict regulations (*t* = 14.772; *p* = 0.000), with the older unemployed in this case being the subjects that held lower levels of fear toward aspects such as not having a flexible timetable, a stressful job or one that limits their free time, or being subject to rigid rules.

On the other hand, in relation to the interrelationships between grieving and the different variables involved in employability, Table 2 shows the correlation coefficients obtained between the different variables in order to determine whether the greater or lesser intensity with which older unemployed people experience grieving is related to their potential for employment. In this sense, we find a positive association between the total grief intensity and the importance that the elderly unemployed give to work, both in terms of the role to be played in life overall (*r* = 0.696; *p* = 0.000), and of other aspects of life (relative centrality, *r* = 0.391; *p* = 0.000). Significant intercorrelations are also found between the grief close to the moment of job loss and that experienced at present.

**Table 2:** Intercorrelations found between the intensity of grief and “employability” measures.

	Total grief	Past grief	Current grief
**Centrality**			
Absolute	0.696^∗∗^	0.686^∗∗^	0.683^∗∗^
Relative	0.391^∗∗^	0.403^∗∗^	0.372^∗∗^
**Work goals**			
Personal development	-0.09	-0.075	-0.161
Professional development	-0.045	-0.050	-0.037
Independence at work	-0.154	-0.097	-0.215
Convenience/comfort	-0.049	-0.050	-0.037
Instrumental index	0.081	0.014	0.001
**Availability**			
DGE	-0.191	-0.196	-0.250
DEF	0.075	-0.061	-0.078
DHR	0.011	-0.077	-0.166
APN	0.795^∗∗^	0.775^∗∗^	0.785^∗∗^
ACP	0.656^∗∗^	0.685^∗∗^	0.621^∗∗^
AVE	0.119	-0.215	-0.209
**Difficulties**			
IRF	0.263^∗^	0.274^∗^	0.249^∗^
IFO	-0.060	-0.076	-0.048
IAT	0.162	0.158	0.161
ICP	0.167	0.170	0.161
IHB	0.197	0.193	0.194
**Fears**			
ENT	0.018	0.002	0.028
ETM	-0.752^∗∗^	-0.758^∗∗^	-0.728^∗∗^
EPS	0.129	0.074	0.159
EDT	0.106	0.093	0.111
EDF	0.075	0.063	0.080
EQM	-0.054	-0.037	-0.063
			


*^∗^*p* < 0.05; ^∗∗^*p* < 0.01.*

Other dimensions with which the intensity of the experience of grieving by older jobseekers positively correlates are the subdimension “family responsibilities” (*r* = 0.263; *p* = 0.043) from the Perceived Difficulties scale, as well as the sub-dimensions “Jobs or tasks I don’t like” (*r* = 0.795; *p* = 0.000) and “Unattractive conditions” (*r* = 0.656; *p* = 0.000) from the Availability scale. Finally, regarding fears, a correlation is found, negative in this case, with the dimensions “Fear of working hard” (*r* = -0.752; *p* = 0000). As for the work goals, there is no interrelation between the experience of grieving and any of the subdimensions.

## Discussion

The current economic recovery in the European Union, after a difficult recession period, has reactivated the creation of jobs at a good pace, even though this binomial economic improvement/increase in employment does not occur at the same rate as in the northern and central European Union countries—where the progress is positive—compared to the countries of the south (Portugal, Italy, Greece and Spain). In the latter, there is an additional phenomenon of long-term structural unemployment that affects almost half of its unemployed population. And in this situation, older workers are the group most affected, with the harmful effects that this socially exclusive situation of long-term unemployment brings to the person who suffers it: loss of self-esteem, lack of motivation and decrease in jobseeking attitudes (Oña, 2014).

In the case of Spain, 757,000 of the 1.2 million people out of work aged 45 to 59 in 2017 were long-term unemployed, which is 63% of the total, a figure much higher than the average long-term unemployment in other age groups. This is compounded by the fact that becoming unemployed for an older worker means a great risk of never being able to return to employment, as the data indicate that over 40% of them will still be out of work 12 months later. Even worse, if they exceed this threshold, the likelihood of remaining unemployed at 24 months is 80% (Bentolila et al., 2017). This difficult escape from unemployment causes many of these jobless older workers to desist from actively seeking employment and become inactive and discouraged. In the study by Bentolila et al. (2017), only 30 out of every 100 older unemployed workers wanted to work and showed enthusiasm for seeking employment. The rest claimed that they had no hope of escaping their unemployed situation, believing that they would never find a job. The results confirmed, moreover, that the probability of escaping unemployment gradually dwindles along with age. Being 10 years older translates as an increase in the unemployment rate after 12 months between 5.4 and 7.4%, with unemployed people with a higher educational level being least likely to enter the labour market, among other reasons, due to their greater reluctance to accept any job and precarious working conditions. The risk of social and economic exclusion among long-term unemployed persons in this age group is thus real and considerable.

However, in addition to considering age as an important factor in the behaviour of individuals toward the job market, the negative consequences of becoming unemployed are greater as the involvement with work increases. Work provides day-to-day activity with a temporal structure, enables the forging of social relationships with other people outside the family nucleus, generates other goals of a social nature, reinforces the development of an activity and defines the identity and status of the person (Jahoda, 1987); in other words, it provides a series of latent functions beyond merely economic resources. The consequences of unemployment would therefore be explained by the loss of these latent functions. This situation is exacerbated when we apply it to certain age groups which, without being considered excluded from the dynamic of insertion into active life, present certain characteristics that make them highly vulnerable, as is the case of older workers.

The first results found in our study, indicating greater intensity and duration of grieving due to job loss in older workers than in other ages, are located in the two lines of argument stated so far. On the one hand, that involvement in the job is related to the worker’s age, and this would indicate a greater impact on the loss of that job the older the worker is; and a second argument that highlights the negative perception of older workers in terms of returning to work, based on the data provided by the labour market.

This involvement with the “thing” lost, the job, as well as the hopelessness of not recovering it again, supposes a situation very similar to “chronic grief” (Bonanno et al., 2004), which identifies that the greater intensity and length of grieving, the greater the dependence and affective bond the person had with the person, thing or situation lost. Moreover, studies such as those of Stroebe and Stroebe (1987) or those of Schulz et al. (2006) have shown that the most vulnerable people are more prone to the development of complications in the process of grieving due to loss, with some of these complications being related to the intensity and prolongation of the grieving process.

On the other hand, the situation of risk of exclusion to which unemployment usually leads older workers, due to the differential features of the group that make it more vulnerable to an exclusionary labour market that causes uncertainty and insecurity, is compounded by the low social visibility of this problem. This occurs despite the harshness of the situation faced daily by the unemployed in this age group, as social and workplace insertion efforts are mainly focused on other groups with greater visibility, such as young people (Centre of Research and Documentation on issues of Economy, Employment and Professional Qualifications [CIDEC], 2012). This lack of social support is corroborated in the data that were gathered in relation to the greater intensity and prolongation of grieving due to job loss in older people. This is one of the protection factors most cited in studies on psychological grief (Sanders, 1999) and, therefore, a risk factor for chronic grief, as when the person suffering the loss feels that they do not have adequate or expected social support, the grief intensifies and becomes prolonged.

Regarding the data obtained that tell us about similar intensity and duration of grief in older workers regardless of the time elapsed since losing the job, agreements were found with most studies on grief, in which no significant differences in the levels of grieving are found (Schwab et al., 1975). However, Prigerson et al. (2002) noted that the symptoms of chronic grief are resolved through time. Nevertheless, many of the studies on intensity and duration of grief were carried out after only short periods of time since the loss, usually only a few months, which might explain the minimal differences observed between groups.

Several studies indicate that the consideration of work as a central value in a person’s life is directly related to age (Lipovetsky, 2008). Thus, Cogin (2012) states that age represents a factor that enables or conditions the way work is conceived, and that this relationship is modulated by specific occupational circumstances (García et al., 2001). Different sectors compare the significance of the work of the younger population, understanding it as a rather frivolous activity and lacking any major importance beyond the strictly material, in contrast to the adult sectors that value it as a personal and collective duty (Morrison et al., 2006; Fenzel, 2013).

As to the significance and centrality of work in unemployed groups, recent studies indicate that unemployed workers present a high degree of work centrality, both absolute and relative, caused precisely by the scarcity or inability to find paid employment. However, the fundamental motivating value is mainly limited to the instrumental objective—in other words, earning income (Izquierdo and Alonso, 2010; Padilla et al., 2012).

This trend toward increased centrality in relation to the age of the person, as well as the verification of high levels of significance given to work in unemployment situation, is related to the outcomes in our study, finding that older unemployed people attribute greater importance to work in global terms. Regarding the relative centrality for the older group, the most important life areas are, in this order, family, work, studies and life partner. Meanwhile, in the other unemployed participants, work appears as the most important area, followed by the life partner, friends and family, on a scale of greater to lesser importance. As noted earlier, the social exclusion situation of older unemployed workers becomes exacerbated by their low social visibility. In general terms, this means little social-institutional support, with few specific initiatives for the insertion of this collective. Along with the phenomenon designated by authors such as Segura and Topa (2016) as “identification with older workers,” and which refers to the internalisation of negative beliefs and attitudes toward older workers by older workers themselves, this causes these people to focus their efforts, emotions and values in the family, in a clear strategy of “social refuge.”

As for the results obtained in relation to the difficulties perceived in the search for employment, and which highlight significant differences among the older workers compared to the rest of the participants in the study, works such as that by Juan et al. (1998) point to the relationship between the information available to individuals about the labour market and the forming of expectations of control over the achievement of employment. They observe a clear relationship between unemployment rates and variables such as the level of information available to the unemployed, the level they believe they have in relation to what they consider necessary to be successful in the job market, or the levels of job placement expectations that the unemployed themselves have. These authors related it to Seligman’s theoretical postulates of learned helplessness, in that it is not necessary to have been subjected to direct experiences of objective uncontrollability to form expectations of not having control over the results. In this sense, it is possible to understand that older workers, among other reasons due to a generalised low perception of self-efficacy in terms of understanding the current job market, as well as the keys and techniques for successful insertion, have internalised this situation by perceiving it as an important obstacle in their search for a job.

On the other hand, García and García (2008) emphasise that there is a significant association between age and preference to accept certain conditions. Thus, the older the unemployed person, the less predisposed they will be to accept precarious situations (with no contract), with lower remuneration than the established norm, or accept part-time jobs. This positioning would influence the older worker’s perception of their possible workplace insertion, as by opting for jobs in proper conditions, they understand that their possibilities are reduced, and much more so in an increasingly precarious market that ousts older workers in order to replace them with younger people whose rights and salaries it curtails.

Moreover, in terms of availability, the older jobseekers in the study would accept jobs that involved working conditions that required high availability, as well as jobs and tasks that they did not like and in unattractive conditions. Again, the scant expectations of finding a job in this collective—due to the adverse job market conditions, little social visibility, lack of institutional support and their own negative self-image regarding their insertion—mean that in the event of our hypothetical job offer, they present high availability, which allow them to adapt to the conditions of the job, even accepting unattractive conditions, although without going so far as to be precarious. These outcomes contrast with those of other studies such as that carried out by Alonso-García (2004b) into what a sample of Spanish unemployed people would accept or reject regarding a job, finding that the older subjects presented lower availability.

Regarding the fears perceived by unemployed people in the jobseeking process and related situations, there were significant differences between older unemployed workers and other jobseekers in the fear of hard work, without set timetables and under strict rules, and in this case it was the older workers who perceived lower levels of fear toward these aspects. According to Alonso-García and Sánchez-Herrero (2011) fear of hard work is usually associated with the need for control. In this sense, the relationship between insecurity and loss of control in the context of work and organisational change, and how this situation comes about, mainly in older workers, is well-known. In short, and as stated by Krumboltz et al. (1976), we must detect these fears, analyse them and intervene if they act as barriers to the individual’s incorporation into the labour market.

Montalbán (1994) points to the positive linear relationship between age and involvement in work, which would explain that the older workers in our study are those with the least fear of working hard, without schedules or under rigid rules. In turn, Mathieu and Zajac (1990) found positive correlation indexes between age and organisational commitment. These data would be in line with some models (Meyer and Allen, 1984) that suggest how older workers increase their commitment for a variety of reasons, including a greater predisposition to positive work attitudes.

Regarding the results in the present study, and which relate grieving due to job loss to the variables that make up the employability of older jobseekers, we found significant evidence that the higher the work centrality presented by the older worker, the more the job loss grieving process intensifies. And this situation comes about whether the older worker values the job as a role to be played in life overall, or in terms of other aspects of life.

Rodríguez-Montalbán et al. (2017) state that work centrality positively predicts intra- and extra-role performance, through harmonious passion, understood as a strong inclination toward an activity which the person likes, which they consider important, which is part of his identity and on which they spend a significant amount of time and energy (Vallerand et al., 2010). That is, the job reflects a part of the identity of the person who deems it important and central to their life, so they develop the inclination toward it (Vallerand, 2015). Following this approach, the more central the job becomes as part of their identity, the more passionate these people will feel toward it (Murnieks et al., 2014) and, in contrast, they will also experience more intense grief at the moment the object of their passion, i.e., the job, is lost.

Another aspect of the relationships brought to light in our study, and which affects older workers in relation to other workers of younger age, is characterised by the intensity of the experience of grief and some perceived difficulties in the search for employment, specifically family responsibilities, accepting positions or tasks that they do not like and accepting jobs with unattractive conditions. An intensive grieving process is similar to a traumatic event that generates anxious-depressive symptoms and gives rise to a profound transformation of the vision of life and the most intimate values of the person who suffers it Echeburúa (2004). It is therefore to be expected that the grieving person will feel little enthusiasm for seeking and accepting work, and even more so if this job is in an activity or post that bears no relation to their professional profile, or involves unattractive or demotivating tasks, or if the conditions associated with this position—such as salary, schedule, category, etc.—are not appealing to the individual.

## Conclusion

Loss of employment and the psychological and health consequences of this situation are identified with those that arise in the grieving process. In this sense, older workers present a series of features that determine that their job loss grieving process is more intense and lasts longer than that of other younger workers, regardless of whether the job loss was recent or not. These characteristics are related to the lack of social support due to the group’s invisibility and the scarcity of public support measures, the negative perception of older workers in terms of assessing their chances of returning to work, as well as the great involvement with work in employees at this age and which is related to an increase in negative consequences in the event of losing the job.

In relation to the analysis of the variables that make up the level of employability in older workers compared to their younger peers, the study shows conclusive data regarding a higher level of work centrality, both relative and absolute in the former with respect to the latter. As for the greater difficulties perceived in jobseeking by the older workers compared to the younger ones, this can be explained, among other reasons, by a low perception of self-efficacy in terms of understanding the current labour market as well as of the keys and techniques to successful insertion.

In terms of availability, the older jobseekers in the study would accept jobs that involved working conditions that required high availability, as well as jobs and tasks that they did not like much and in unattractive conditions, which may be explained by their scant expectations of finding work. In relation to the perceived fears, it is the older workers who hold higher levels of fear of working hard, without schedules and with rigid rules.

As to the results that relate grieving for job loss to the variables that conform the employability of older jobseekers, we found significant evidence that the greater the work centrality manifested by the older worker, the more the job loss grieving process intensifies. This may be explained by the foreseeable relationship of centrality with the passion for work, with the loss of employment being felt more intensely in these circumstances.

Finally, the study found significant relationships between the intensity of grief and the perceived difficulties in finding a job: family responsibilities, accepting jobs or tasks that they do not like and accepting jobs with unattractive conditions.

### Limitations

The main limitation of this study consisted of recruiting a sufficient number of people to make up a significant sample. Unemployed people in general, in many cases, suffer a process of discouragement and burnout, which in many cases has led to mistrust of employment services, as they understand that they have been unable to respond to their needs. Moreover, they are submerged in very complex bureaucratic processes that only impose an additional burden on their psychological state. The feeling of abandonment by the administrations and the need to obtain a job, or a decent job, are their main objectives. So, the willingness to take part in this type of research, in many cases, requires extra effort from the unemployed as well as the researchers. On the other hand, coping with grief has always been analysed from the perspective of the loss of a loved one. It is very difficult to find studies that address this process in terms of job loss, in order to draw exhaustive comparisons with other groups.

## Author Contributions

JC-R and YN-A conceived and designed the study, with the assistance of ML-L, JG-S, and MG. YN-A and ML-L carried out the analyses, with contributions from all the other authors. The main versions of the manuscript were written by JC-R, with contributions from all the other authors. All the authors participated in the interpretation of the results and approved the final version and are jointly responsible for an appropriate review and discussion of all aspects included in the manuscript.

## Conflict of Interest Statement

The authors declare that the research was conducted in the absence of any commercial or financial relationships that could be construed as a potential conflict of interest.
